# Supplementary material for: Rabies control via co-creation: A model for sustainable one health interventions
Source: PLoS Negl Trop Dis. 2025 Aug 11;19(8):e0013350. doi: 10.1371/journal.pntd.0013350 (PMC12517478; doi:10.1371/journal.pntd.0013350)
Supplement: Attachment — Submitted filename: 2025-04-11_PNTD-D-25-00145_Review.docx [file pntd.0013350.s001.docx]

**Summary**

This manuscript presents findings from a co-creation strategy aimed at developing communication materials for rabies awareness and mass dog vaccination campaigns (MDVC). The study makes a valuable contribution to the rabies literature by addressing a critical, yet often overlooked, barrier to successful MDVC implementation—namely, communication challenges and insufficient community engagement. By involving community stakeholders in the development of communication tools, the study offers a practical and context-sensitive approach to improving public health interventions. I recommend this manuscript for publication, pending minor revisions that I believe will strengthen the clarity and overall impact of the work.

**General comments**

-Not all readers will be familiar with jargon associated with qualitative epidemiology / sociology. Please make sure you clearly define any introduced jargon (sensitization, knowledge suppliers, knowledge brokers etc.)

-Many areas of the methods section could use clarification/elaboration:

1) the first co-creation workshop methodology was described in detail but the second only mentioned. Please provide some more detail on the methodology for the second co-creation workshop;

2) please give more detail in the deductive thematic analysis approach methods, including the coding and theme summarization process;

3) Were all interviews in English or were interviews available Kikamba/Kiswahili? If all were in English, please justify this decision and address in the discussion how this may have biased results. If interviews were conducted in multiple languages, please include the translation process in the ‘data transcription and analysis’ section of the methods;

4) a map, if available, would help readers fully understand the study site.

- include intext citations to all figures. Add more description to the figure captions.

-you mention in the abstract that a series of free vaccination drives were conducted 2021-2024, with this study being conducted in 2021 prior to initiation of the drives. Is there any data about participation in the drives?

**Minor comments** (by numbered line)

(39) should read “caused by **a** *lyssavirus*”

(40) Consider changing first line to reflect that all mammals are susceptible to rabies with your second sentence clarifying that dogs are the major source of transmission to humans. As it reads now, it may be confusing to some PLOS NTDs readers from areas where wildlife are the major vector if they are unfamiliar to the global rabies situation

(43) & (50) the term ‘elimination’ instead of ‘eradication’ is more correct when speaking of regional elimination of a pathogen

(49) Please give some context to the Zoonotic Disease Unit. Is this a federal government agency? A regional government agency? A unit associated with a university or other institute?

(57) please define sensitization in this context

(59) please give some context to the One Health High Level Expert Panel (ie explain it is the One Health advisory group to WHO/WOAH/FAO/UNEP)

(60) steinclusivity – typo?

(66) please define knowledge brokers and knowledge suppliers

(Fig 1) review the colors of the background / arrows. The dark blue arrows are difficult to see on the black background.

(101) were rankings made as a group or individually?

(142) in text citation to figure 7 – did you mean figure 6?

(316) typo – should read “…. which **is** below the recommended 70%...”

(321) type – picked?

(322) run-on sentence

(336) should read “… **more** sustainable than….”

(337) please contextualize why cost-effectiveness analysis are important in relation to the co-creation strategy.
