# Supplementary material for: Rabies control via co-creation: A model for sustainable one health interventions
Source: PLoS Negl Trop Dis. 2025 Aug 11;19(8):e0013350. doi: 10.1371/journal.pntd.0013350 (PMC12517478; doi:10.1371/journal.pntd.0013350)
Supplement: Attachment — Submitted filename: PNTD-D-25-00146_review.docx [file pntd.0013350.s002.docx]

PNTD-D-25-00146

Title: Rabies Control via Co-Creation: A Model for Sustainable One Health Interventions

This research approaches to develop a theoretical model to understand the value of dogs to the owners and co-create rabies sensitization health risk communication materials to enhance vaccination uptick. The researchers performed key-informant interviews, focused group discussions, and a co-creation workshop. Results from key-informant interviews were presented during the focused group discussions and pairwise ranking were performed on the dog ownership values. This was followed by co-creation workshop to develop rabies sensitization materials. The research identified securing households, livestock, and crops as a tangible role of dogs in the community and friendship was highlighted as the most important intangible role. The co-creation of rabies sensitization materials involved reflecting values of dogs to the communities, cultural appropriateness and community insights.

The authors recommended integrating community insights and co-create tailored solution before implementing health interventions as it enhances community participation, foster trust and promote sustainability.

**Major comments:**

The author's recommendation for incorporating co-creating health risk communication materials into public health programs seems premature and out of scope of this research due to several reasons. First, there is no quantification provided regarding how much additional percentage of vaccine uptake could be achieved through this process and its associated cost-benefits to evaluate intervention feasibility. Second, most countries already tailor health risk communication materials for regional cultural sensitivity in their systems; therefore, part of this process is already embedded within the system itself. Finally, the study participants for FGD and co-creation workshop were selected from the communities who presented their dogs to vaccination sites, which could have introduced a selection bias; thus, generalizing the findings might be an issue (Note: Corrected grammatical inconsistency).

**Minor comments:**

1. L 153: “24 were interviewed”, please break this number by gender
2. L 197 – L 200: Tables 1 and 2 consists of ‘value numbers’ and ‘scores’ by both male and female participants. However, definition of the ‘value numbers’ and how the ‘scores’ were calculated were not described in the methods.
3. There is an inconsistency in characterizing the participants, e.g., L 254, L 295, L 298, L300.
4. Conclusions need to be consistent with the study findings.
