# Supplementary material for: Rabies control via co-creation: A model for sustainable one health interventions
Source: PLoS Negl Trop Dis. 2025 Aug 11;19(8):e0013350. doi: 10.1371/journal.pntd.0013350 (PMC12517478; doi:10.1371/journal.pntd.0013350)
Supplement: Attachment — Submitted filename: Response to Reviewers.docx [file pntd.0013350.s003.docx]

**RESPONSE TO EDITORS – REBUTTAL LETTER**

Manuscript: Rabies control via Co-creation: A Model for Sustainable One Health Interventions

Dear Editors,

Thank you for the opportunity to revise and resubmit our manuscript to *PLOS Neglected Tropical Diseases*. We appreciate the comments, suggestions, and feedback by the reviewers and the editorial team. This has improved the quality of manuscript. We have carefully reviewed and incorporated the suggestions and we believe it now meets the journal standard. A detailed summary of the changes made in response to each reviewer’s comments is provided in the table below.

We have also included an Author Summary between the Abstract and Introduction sections of the manuscript. This summary is written in accessible language to ensure the findings are understandable to a broader audience, including readers outside the immediate research field.

All main figures have been saved and submitted as separate high-resolution .tif files, following the journal’s formatting specifications. We confirm that Geoffrey Njenga, a co-author, is the photographer for Figures 1, 2, 6, and 8. Annabel Slater, also a co-author, hand-drew Figures 3, 4, 5, 7, and elements of Figure 8 (specifically Figures 8a and 8b). In line with PLOS copyright requirements, we have removed all logos or branding from Figures 3, 4, 5, 7, and 8.

We are grateful for your continued consideration of our manuscript and trust that the revised version will meet the expectations of *PLOS Neglected Tropical Diseases*. Please do not hesitate to contact us should any further clarifications be required.

Sincerely,
Nicholas Bor
University of Edinburgh
On behalf of all co-authors

**RESPONSE TO REVIEWERS**

| **Title: Rabies Control via Co-Creation: A Model for Sustainable One Health Interventions** | |
| --- | --- |
| **Reviewer 1: PNTD-D-25-00146** | |
| **General Comments**  This research approaches to develop a theoretical model to understand the value of dogs to the owners and co-create rabies sensitization health risk communication materials to enhance vaccination uptake. The researchers performed key-informant interviews, focused group discussions, and a co-creation workshop.  Results from key-informant interviews were presented during the focused group discussions and pairwise ranking were performed on the dog ownership values. This was followed by co-creation workshop to develop rabies sensitization materials. The research identified securing households, livestock, and crops as a tangible role of dogs in the community and friendship was highlighted as the most important intangible role. The co-creation of rabies sensitization materials involved reflecting values of dogs to the communities, cultural appropriateness and community insights.  The authors recommended integrating community insights and co-create tailored solution before implementing health interventions as it enhances community participation, foster trust and promote sustainability. | **Authors’ Comments & Responses**  Thank you for taking time to review the paper. The comments raised have been useful in improving the manuscript.  The comments and suggestions have been addressed and appropriate changes made in the revised manuscript. We have included the line numbers of where the changes have been made for easier cross-referencing. |
| **Major Comments**  The author's recommendation for incorporating co-creating health risk communication materials into public health programs seems premature and out of scope of this research due to several reasons. First, there is no quantification provided regarding how much additional percentage of vaccine uptake could be achieved through this process and its associated cost-benefits to evaluate intervention feasibility. Second, most countries already tailor health risk communication materials for regional cultural sensitivity in their systems; therefore, part of this process is already embedded within the system itself. Finally, the study participants for FGD and co-creation workshop were selected from the communities who presented their dogs to vaccination sites, which could have introduced a selection bias; thus, generalizing the findings might be an issue  (Note: Corrected grammatical inconsistency). | **Response**  Thank you for your comments and observations.  Your point on the bias within the study population is well noted. We have specifically identified this as a limitation in the manuscript (Lines 394 – 397).  We premised our work on the assumption that community engagement is a positive step for public health interventions as widely recognized. We therefore undertook the current study specifically to trial a mechanism for incorporating community insights into sensitization material for rabies vaccination campaigns.  Whilst we agree that a quantitative evaluation of this would have been beneficial, we did not set out to measure the additional vaccine uptake this will result.  We also acknowledge that while tailored messages are becoming more regularly incorporated in health intervention programs, we have noted is not always the case in all places. An example is WASH project in India that failed to include community insight and didn’t succeed as expected as there was lack of buy-in and project ownership by the community. Lines –383 – 386. |
| **Minor Comments**  L153: “24 were interviewed”, please break this number by gender | The number of participants attending the planned research activities have been broken by gender as suggested. Line 204 |
| L197 – L 200: Tables 1 and 2 consists of ‘value numbers’ and ‘scores’ by both male and female participants. However, definition of the ‘value numbers’ and how the ‘scores’ were calculated were not described. | We understand that the ‘value number’ assigned in pairwise ranking table may confuse the reader.  We have now re-assigned the dog values as alphabets (rather than numbers) to avoid confusing the reader. We have elaborated in the methods section how pairwise ranking was done in the revised manuscript. Lines 247 – 249. |
| There is an inconsistency in characterizing the participants, eg., L254, L 295 | We have addressed the inconsistency in characterizing the participants by addressing them as male and female respondents throughout the manuscript. |
| Conclusion needs to be consistent with study findings | We have revised the conclusion to include key study findings—particularly the roles and values that dogs hold in the community.  It now reflects how these findings influenced community engagement, increased awareness, and improved motivation to vaccinate, thereby strengthening the recommendation on the need to incorporate community insights during health interventions. Lines 405 -418. |
| **Reviewer 2: N/A** | |
| No comments | |
| **Reviewer 3: PNTD-D-25-00145** | |
| **General Comments**  This manuscript presents findings from a co-creation strategy aimed at developing communication materials for rabies awareness and mass dog vaccination campaigns (MDVC). The study makes a valuable contribution to the rabies literature by addressing a critical, yet often overlooked, barrier to successful MDVC implementation—namely, communication challenges and insufficient community engagement.  By involving community stakeholders in the development of communication tools, the study offers a practical and context-sensitive approach to improving public health interventions. I recommend this manuscript for publication, pending minor revisions that I believe will strengthen the clarity and overall impact of the work. | **Authors’ Responses**  Thank you for the positive comments and in seeing the value of co-creating context specific materials for public health initiatives. We are glad that the approach we are suggesting to be appropriate and something you can apply in your rabies projects.  Your reviews and comments have been valuable in strengthening the clarity and impact of this study. |
| **Major comments**  Many areas of the methods section could use clarification/elaboration:  Not all readers will be familiar with jargon associated with qualitative epidemiology / sociology. Please make sure you clearly define any introduced jargon (sensitization, knowledge suppliers, knowledge brokers etc.) | We have clarified the methods section and removed or explained "jargon" where appropriate. |
| The first co-creation workshop methodology was described in detail but the second only mentioned. Please provide some more detail on the methodology for the second co-creation workshop; | More details of the 2nd co-creation workshop have been added - Lines 149– 152.  We have also added subtitles of the 1^st^ and 2^nd^ co-creation workshops to guide the reader. Lines 156 and 183 respectively. |
| Please give more detail in the deductive thematic analysis approach methods, including the coding and theme summarization process | This has been expanded and the relevant citation added. Lines 125 – 128. |
| Were all interviews in English or were interviews available Kikamba/Kiswahili? If all were in English, please justify this decision and address in the discussion how this may have biased results. If interviews were conducted in multiple languages, please include the translation process in the ‘data transcription and analysis’ section of the methods | There was no specific language requirement. Respondents were interviewed in the language that they were most comfortable with. This ensured delivery of quality responses. This has been explained in lines 113 – 118. |
| A map, if available, would help readers fully understand the study site. Include in-text citations to all figures. Add more description to the figure captions. | The figures have been updated within the text for easier co-referencing.  The figure captions describing each activity in the photo grid have been added.  We have developed a map of the study site and will be incorporated in the revised manuscript. |
| You mention in the abstract that a series of free vaccination drives were conducted 2021-2024, with this study being conducted in 2021 prior to initiation of the drives. Is there any data about participation in the drives? | This study was conducted in 2023 and there was no prior data on the level of community participation in this study cite.  However, a similar vaccination campaign within Kenya found 24% community engagement and this may be extrapolated to our study site  <https://doi.org/10.1371/journal.pntd.0008260> |
| **Minor Comments**  L39 Should read “caused by a lyssavirus” | This has been updated – Line 56. |
| L40 Consider changing first line to reflect that all mammals are susceptible to rabies with your second sentence clarifying that dogs are the major source of transmission to humans. As it reads now, it may be confusing to some PLOS NTDs readers from areas where wildlife is the major vector if they are unfamiliar to the global rabies situation | This statement is now updated as suggested – Line 56. |
| L43 & L50 the term ‘elimination’ instead of ‘eradication’ is more correct when speaking of regional elimination of a pathogen | Updated as suggested – Lines 62 and 70 respectively. |
| L49 Please give some context to the Zoonotic Disease Unit. Is this a federal government agency? A regional government agency? A unit associated with a university or other institute? | ZDU is a government agency which is a One Health office in Kenya.  I have provided the context for the reader in the revised manuscript for clarity. (Lines 66 -69). |
| L57 Please define sensitization in this context | The jargon (sensitization) has been explained in the revised manuscript - Line 76. |
| L59 Please give some context to the One Health High Level Expert Panel (ie explain it is the One Health advisory group to WHO/WOAH/FAO/UNEP) | Context has been provided for clarity - Line 79. |
| L60 steinclusivity – typo? | Typo has been corrected - Line 81. |
| L66 Please define knowledge brokers & knowledge suppliers | This sentence has been reworded into a simpler sentence without the jargons in the revised manuscript. Lines 82 – 88. |
| (Fig 1) review the colors of the background / arrows. The dark blue arrows are difficult to see on the black background. | We have developed and uploaded a new figure where the arrows in the photo grid are visible. |
| L101 Were rankings made as a group or individually? | It was done collectively as a group. That clarification has been added on the revised manuscript - Line 134 – 141. |
| L142 in text citation to figure 7 – did you mean figure 6? | Thanks for identifying the error. That is now corrected – Line 192. |
| L316 typo – should read “…. which is below the recommended 70%...” | The omission has been included - Line 363. |
| L321 type – picked? | This has been edited to read clearly – Line 368. |
| L322 run-on sentence | The run on sentences have been punctuated corrected. Lines 368 – 370. |
| L336 should read “… more sustainable than….” | Thank you for identifying the error. This is now corrected - Line 383. |
| L337 please contextualize why cost-effectiveness analysis are important in relation to the co-creation strategy. | This has been explained in Lines 389- 391. It is cheaper to vaccinate dogs against rabies that offer post exposure prophylaxis to dog bite victims. Like in this case, the campaign was offering free rabies vaccination meaning the owners and their neighbors are protected. |
